# Outcomes by Cardiac Stage in Patients With Newly Diagnosed AL Amyloidosis: Phase 3 ANDROMEDA Trial

**DOI:** 10.1016/j.jaccao.2022.08.011

**Published:** 2022-11-15

**Authors:** Monique C. Minnema, Angela Dispenzieri, Giampaolo Merlini, Raymond L. Comenzo, Efstathios Kastritis, Ashutosh D. Wechalekar, Martha Grogan, Ronald Witteles, Frederick L. Ruberg, Mathew S. Maurer, NamPhuong Tran, Xiang Qin, Sandra Y. Vasey, Brendan M. Weiss, Jessica Vermeulen, Arnaud Jaccard

**Affiliations:** a University Medical Center Utrecht, Utrecht, the Netherlands; b Mayo Clinic, Rochester, Minnesota, USA; c Amyloidosis Research and Treatment Center, Fondazione IRCCS Policlinico San Matteo, Pavia, Italy; d Department of Molecular Medicine, University of Pavia, Pavia, Italy; e John C. Davis Myeloma and Amyloid Program, Tufts Medical Center, Boston, Massachusetts, USA; f National and Kapodistrian University of Athens, Athens, Greece; g University College London, London, United Kingdom; h Stanford Amyloid Center, Stanford University School of Medicine, Stanford, California, USA; i Boston Medical Center, Boston University School of Medicine, Boston, Massachusetts, USA; j Columbia University Irving Medical Center, New York, New York, USA; k Janssen Research & Development LLC, Los Angeles, California, USA; l Janssen Research & Development LLC, Spring House, Pennsylvania, USA; m Janssen Research & Development LLC, Leiden, the Netherlands; n Centre Hospitalier Universitaire and Reference Center for AL Amyloidosis, Limoges, France

**Keywords:** daratumumab, Mayo staging system, AE, adverse event, AL, amyloid light chain, CR, complete response, D-VCd, daratumumab, bortezomib, cyclophosphamide, and dexamethasone, EFS, event-free survival, FLC, free light chain, hs-cTnT, high-sensitivity cardiac troponin T, NT-proBNP, N-terminal pro–brain natriuretic peptide, NYHA, New York Heart Association, PFS, progression-free survival, SAE, serious adverse event, VCd, bortezomib, cyclophosphamide, and dexamethasone

## Abstract

**Background:**

Patients with amyloid light chain amyloidosis and severe cardiac dysfunction have a poor prognosis. Treatment options that induce rapid and deep hematologic and organ responses, irrespective of cardiac involvement, are needed.

**Objectives:**

The aim of this study was to evaluate the impact of baseline cardiac stage on efficacy and safety outcomes in the phase 3 ANDROMEDA trial.

**Methods:**

Rates of overall complete hematologic response and cardiac and renal response at 6 months and median major organ deterioration–progression-free survival and major organ deterioration–event-free survival were compared across cardiac stages (I, II, or IIIA) and treatments (daratumumab, bortezomib, cyclophosphamide, and dexamethasone [D-VCd] or bortezomib, cyclophosphamide, and dexamethasone [VCd]). Rates of adverse events (AEs) were summarized for patients with and without baseline cardiac involvement and by cardiac stage.

**Results:**

Median follow-up duration was 15.7 months. The proportions of stage I, II, and IIIA patients were 23.2%, 40.2%, and 36.6%. Across cardiac stages, hematologic and organ response rates were higher and major organ deterioration–progression-free survival and major organ deterioration–event-free survival were longer with D-VCd than VCd. AE rates were similar between treatments and by cardiac stage; serious AE rates were higher in patients with cardiac involvement and increased with increasing cardiac stage. The incidence of cardiac events was numerically greater with D-VCd vs VCd, but the rate of grade 3 or 4 events was similar. The exposure-adjusted incidence rate for cardiac events was lower with D-VCd than VCd (median exposure 13.4 and 5.3 months, respectively).

**Conclusions:**

These findings demonstrate the efficacy of D-VCd over VCd in patients with newly diagnosed amyloid light chain amyloidosis across cardiac stages, thus supporting its use in patients with cardiac involvement. (NCT03201965)

Systemic amyloid light chain (AL) amyloidosis is a rare clonal plasma cell disease associated with amyloid deposition within vital organs (particularly the heart and kidneys), leading to progressive organ dysfunction and death.^1^ Cardiac involvement in AL amyloidosis manifests as a restrictive cardiomyopathy resulting in congestive heart failure and arrhythmias. The extent of cardiac involvement at baseline is the most important predictor of clinical outcomes,^2^, ^3^, ^4^, ^5^, ^6^, ^7^, ^8^ with median survival of <1 year in untreated patients with severe cardiac involvement vs about 8 years in those without.^5^^,^^9^ Approximately one-third of early deaths (ie, within 90 days of diagnosis) among patients with AL amyloidosis are attributed to cardiac involvement.^10^

Mayo Clinic researchers established a staging system for AL amyloidosis on the basis of the prognostic biomarkers serum high-sensitivity cardiac troponin T (hs-cTnT) and N-terminal pro–brain natriuretic peptide (NT-proBNP).^7^ Patients are classified in stage I if levels of both hs-cTnT and NT-proBNP are less than their respective thresholds (54 ng/L for hs-cTnT and 332 pg/mL for NT-proBNP), stage II if the level of either biomarker is greater than its threshold, and stage III if levels of both biomarkers are greater than their respective thresholds.^11^ Median overall survival for patients in stages I, II, and III was 69, 29, and 6 months, respectively. The European modification of the Mayo staging system further stratified stage III into subgroups: NT-proBNP levels of <8,500 ng/L (stage IIIA) and >8,500 ng/L (stage IIIB).^4^, ^5^, ^6^ Stage III patients are considered at high risk and have an especially poor prognosis, with high rates of early death within months of diagnosis.^2^^,^^4^, ^5^, ^6^

Until recently, standard treatment for AL amyloidosis included off-label use of modified bortezomib-based regimens approved for the treatment of multiple myeloma,^1^^,^^6^^,^^12^^,^^13^ including the combination of bortezomib, cyclophosphamide, and dexamethasone (VCd), which has led to improved outcomes compared with earlier treatment options.^6^^,^^14^^,^^15^ A recent study suggests that patients with severe cardiac involvement at baseline have not experienced the same level of benefit as the overall population of patients with AL amyloidosis,^16^ and there is a need for additional treatment options that will improve outcomes in this high-risk group.

ANDROMEDA (NCT03201965) is a randomized, open-label, active-controlled, phase 3 study examining the safety and efficacy of subcutaneous daratumumab (a human immunoglobulin Gκ CD38-targeting monoclonal antibody), in combination with VCd (D-VCd) compared with VCd alone in patients with newly diagnosed AL amyloidosis. The primary results demonstrated that patients treated with D-VCd achieved deeper and more rapid hematologic responses and higher rates of organ responses than those treated with VCd alone.^17^ The safety profile was consistent with previous studies of daratumumab and VCd.^17^ On the basis of these results, D-VCd became the first treatment for AL amyloidosis to receive regulatory approval.^18^ Here, we evaluate the impact of patients’ cardiac stage on efficacy and safety outcomes in patients from ANDROMEDA.

## Methods

### Patients and design

The primary report of ANDROMEDA has been published.^17^ In brief, ANDROMEDA is a randomized, open-label, active-controlled, multicenter, phase 3 study in patients with newly diagnosed AL amyloidosis (NCT03201965). Each study site’s local independent ethics committee or Institutional Review Board approved the study protocol. The study was conducted in accordance with the principles of the Declaration of Helsinki and the International Conference on Harmonisation of Technical Requirements for Pharmaceuticals for Human Use Good Clinical Practice guidelines. All patients provided written informed consent.

Patients were randomized in a 1:1 ratio to receive 6 cycles (28 days each) of D-VCd or VCd. All patients received subcutaneous bortezomib (1.3 mg/m^2^ weekly), oral or intravenous cyclophosphamide (300 mg/m^2^ weekly; maximum weekly dose 500 mg), and oral dexamethasone (20-40 mg weekly). Subcutaneous daratumumab (1,800 mg coformulated with recombinant human hyaluronidase PH20 in 15 mL) was administered weekly in cycles 1 and 2 and every 2 weeks in cycles 3 to 6. After cycle 6, patients in the VCd group completed study treatment, and those in the D-VCd group received subcutaneous daratumumab as their only treatment every 4 weeks until major organ deterioration or death, for a maximum of 24 cycles in total.

Key eligibility criteria included newly diagnosed AL amyloidosis with measurable hematologic disease, ≥1 involved organ, cardiac stages I to IIIA (per the European modification of the Mayo staging system), estimated glomerular filtration rate ≥20 mL/min/1.73 m^2^, and no prior diagnosis of symptomatic multiple myeloma. Key exclusion criteria included NT-proBNP >8,500 ng/L, cardiac stage IIIB (per the European modification of the Mayo staging system), New York Heart Association (NYHA) functional class IIIB (comfortable at rest, shortness of breath with performance of activities of daily living) and functional class IV (shortness of breath at rest, unable to carry out any physical activity without discomfort, signs or symptoms of heart failure or anginal syndrome may be present at rest, discomfort increases with physical activity), and evidence of significant cardiovascular conditions.

### Assessments

In this post hoc analysis, the primary endpoint was the overall rate of hematologic complete response (CR), defined as normalization of free light chain (FLC) levels and FLC ratio and negative serum and urine immunofixation. If involved FLC was lower than the upper limit of normal, normalization of uninvolved FLC level and FLC ratio were not required to define CR.^19^ Hematologic responses were evaluated every 4 weeks for cycles 1 to 6 and every other month thereafter until major organ deterioration–progression-free survival (PFS), death, withdrawal of consent to participate, or the end of the study. Responses were adjudicated by an independent review committee. Secondary endpoints were major organ deterioration–PFS, major organ deterioration–event-free survival (EFS), organ response rate, time to hematologic response, overall survival, and safety.

Major organ deterioration–PFS is a composite endpoint defined as end-stage cardiac disease (requiring cardiac transplantation, left ventricular assist device, or intra-aortic balloon pump), end-stage renal disease (requiring hemodialysis or renal transplantation), hematologic progression per consensus guidelines,^20^ or death (whichever came first). Major organ deterioration–EFS was defined as hematologic progression, major organ deterioration, initiation of subsequent non-cross-resistant anti–plasma cell therapy, or death (whichever came first). This endpoint is similar to major organ deterioration–PFS but also includes the initiation of subsequent non-cross-resistant therapy; it was used to reflect the treatment paradigm in AL amyloidosis. Patients with suboptimal hematologic response or worsening of organ function were allowed to switch to subsequent non-cross-resistant anti–plasma cell therapy before hematologic disease progression or major organ deterioration occurred.

Cardiac and renal response rates were calculated and defined as the proportion of baseline organ-evaluable patients who achieved a response at 6 months. Cardiac response was based on NT-proBNP response (>30% and >300 ng/L decrease in patients with baseline NT-proBNP ≥650 ng/L) or NYHA functional class response (>2-class decrease in patients with baseline NYHA functional class IIIA) per 2012 consensus criteria.^20^ Conversely, cardiac progression rate was based on NT-proBNP progression (>30% and >300 ng/L increase), cardiac troponin T progression (≥33% increase), or ejection fraction progression (≥10% decrease) per 2012 consensus criteria^20^ in the absence of renal progression. Renal response was defined as ≥30% decrease in proteinuria or proteinuria decrease to <0.5 g/24 h in the absence of renal progression (≥25% decrease in estimated glomerular filtration rate), as developed by a group of international experts.^21^

Adverse events (AEs) and laboratory values were assessed. Biomarker assessments were performed centrally.

### Statistical analysis

Analyses of hematologic CR and major organ deterioration–PFS were performed on the intent-to-treat analysis set. Cardiac response analyses were based on patients who were evaluable for cardiac response (ie, patients with baseline NT-proBNP ≥650 ng/L or baseline NYHA functional class IIIA who received ≥1 administration of study treatment). Renal responses were analyzed in patients with baseline urine protein >0.5 g/d.^21^ Patients without baseline or postbaseline assessments were censored at randomization for the major organ deterioration–PFS analysis. The data for hematologic CR, organ response rate, time to hematologic response, overall survival, and safety were based on the clinical cutoff of June 2020, and data for major organ deterioration–PFS and major organ deterioration–EFS used the clinical cutoff of February 2020.

The stratified Cochran-Mantel-Haenszel test with ORs and 95% CIs was used to estimate treatment differences in overall hematologic CR rate as per the main trial design. The stratification factors used in the analysis were cardiac stages (I, II, and IIIA), countries offering transplantation for patients with AL amyloidosis, and renal function (creatinine clearance ≥60 mL/min or <60 mL/min). Descriptive statistics are reported using number, mean, SE, median, and range for continuous variables and frequencies and percentages for categorical variables. The Kaplan-Meier method was used for descriptive summaries of time-to-event endpoints, including major organ deterioration–PFS and cardiac events. Kaplan-Meier curves were plotted for major organ deterioration–PFS by cardiac stage. The stratified Cochran-Mantel-Haenszel test with ORs and 95% CIs was used to assess treatment differences in the proportion of patients with cardiac response at 6 months. A similar analysis was performed for renal response rate. All randomized patients were included in the denominator for the calculation of hematologic CR rates; patients who died without achieving hematologic CR were also included in the denominator. However, patients who died after achieving hematologic CR were included in both the numerator and denominator. Fine and Gray’s method was used to estimate the time to first onset of cardiac event in both treatment groups.

Safety data and exposure were evaluated in the safety population (all randomized patients who received ≥1 administration of study treatment); the safety analysis set included data from the randomized parts of the study. Exposure-adjusted evaluation of AEs was performed because of the longer median duration of exposure in the D-VCd group compared with the VCd group. Statistical analyses were performed using SAS version 9.4 (SAS Institute).

## Results

A total of 388 patients were randomized to D-VCd (n = 195) or VCd (n = 193). Baseline characteristics were well balanced between the 2 groups. The proportions of patients in cardiac stages I, II, and IIIA at baseline were 23.2%, 40.2%, and 36.6%, respectively. Of note, 8 patients progressed from stage IIIA to stage IIIB between screening and the start of treatment. As they were in stage IIIA at screening and met the inclusion criteria, these patients are included in the subgroup referred to as stage IIIA in this report. Median treatment duration was 13.4 months for D-VCd and 5.3 months for VCd. Median follow-up duration was 15.7 months (range: 0.0-24.1 months).

Baseline characteristics were generally well balanced across cardiac stages (Table 1), with some exceptions. As expected, patients with cardiac stage IIIA disease had worse Eastern Cooperative Oncology Group performance status, more advanced renal dysfunction, and functionally worse heart failure (NYHA functional class IIIA). These patients also had the highest mean difference between involved and uninvolved FLC, suggestive of a higher disease burden.

**Table 1:** Baseline Characteristics by Cardiac Stage

	Stage I (n = 90)	Stage II (n = 156)	Stage IIIA^a^ (n = 142)
Age, y	60.5 (35-81)	62.5 (34-86)	66.5 (40-87)
≥65 y, %	30 (33.3)	67 (42.9)	86 (60.6)
Male	57 (63.3)	79 (50.6)	89 (62.7)
ECOG PS^b^			
0	61 (67.8)	63 (40.4)	37 (26.1)
1	28 (31.1)	84 (53.8)	80 (56.3)
2	1 (1.1)	9 (5.8)	25 (17.6)
Number of organs involved	1 (1-4)	2 (1-5)	2 (1-6)
≥2 organs	36 (40.0)	114 (73.1)	104 (73.2)
NYHA functional class			
I	82 (91.1)	79 (50.6)	34 (23.9)
II	8 (8.9)	73 (46.8)	85 (59.9)
IIIA^c^	0	4 (2.6)	23 (16.2)
Renal function status			
CrCl <60 mL/min	21 (23.3)	47 (30.1)	63 (44.4)
Renal stage			
I	41 (45.6)	93 (59.6)	74 (52.9)
II	43 (47.8)	42 (26.9)	56 (40.0)
III	6 (6.7)	21 (13.5)	10 (7.1)
dFLC, mg/L	131.1 (1-4,749)	189.9 (4-9,983)	267.9 (30-4,567)
Isotype of AL amyloidosis			
Kappa	27 (30.0)	31 (19.9)	23 (16.2)
Lambda	63 (70.0)	125 (80.1)	119 (83.8)

Values are median (IQR) or n (%).

AL = amyloid light chain; CrCl = creatinine clearance; D-VCd = daratumumab, bortezomib, cyclophosphamide, and dexamethasone; dFLC = difference between involved and uninvolved free light chain; ECOG PS = Eastern Cooperative Oncology Group performance status; NYHA = New York Heart Association; VCd = bortezomib, cyclophosphamide, and dexamethasone.

a Includes 8 patients (2 in the D-VCd group, 6 in the VCd group) who were in stage IIIA at screening and converted to stage IIIB at cycle 1, day 1 (results determined by central laboratory were made available only after cycle 1, day 1).

b ECOG PS is scored on a scale from 0 to 5, with 0 indicating no symptoms and higher scores indicating increasing disability.

c Patients who were comfortable at rest; less than ordinary physical activity resulted in fatigue, palpitation, dyspnea, or anginal pain.

**Figure 1:**
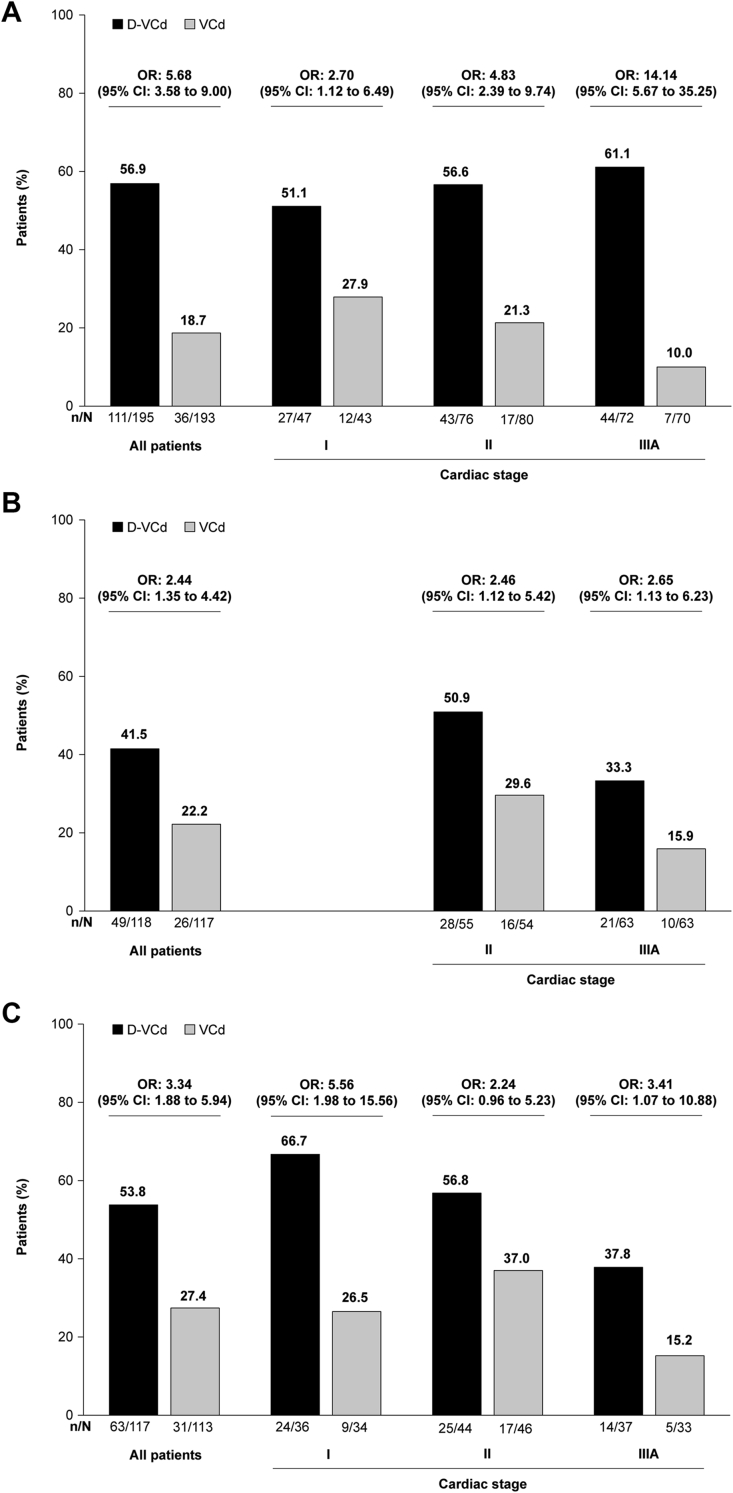
Hematologic CR, Cardiac Response, and Renal Response Irrespective of cardiac stage, rates of overall hematologic complete response **(A)**, cardiac response **(B)**, and renal response **(C)** at 6 months were higher with daratumumab, bortezomib, cyclophosphamide, and dexamethasone (D-VCd) than bortezomib, cyclophosphamide, and dexamethasone (VCd).

**Figure 2:**
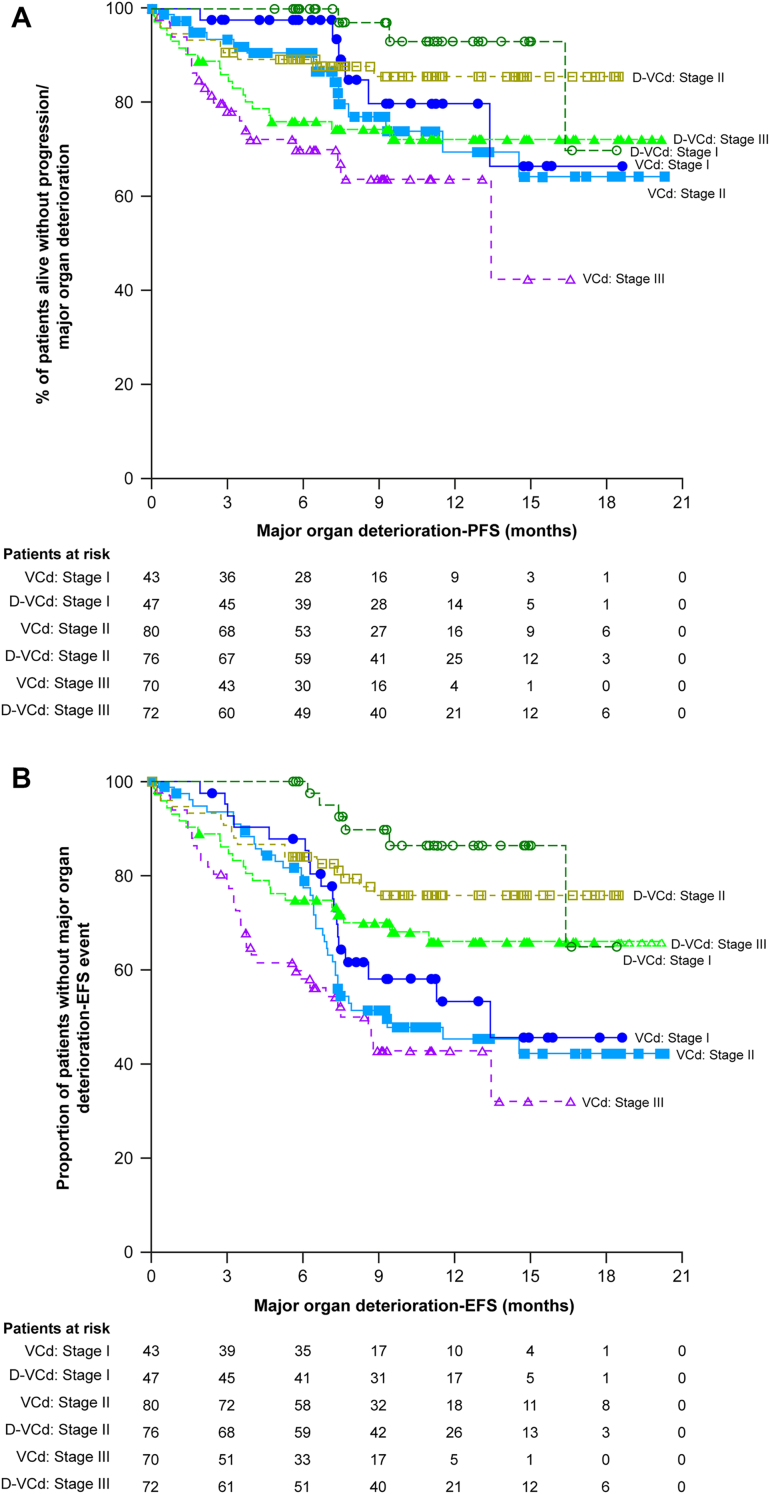
Major Organ Deterioration–PFS and Major Organ Deterioration–EFS Irrespective of cardiac stage, major organ deterioration–progression-free survival (PFS) **(A)** and major organ deterioration–event-free survival (EFS) **(B)** were longer with D-VCd than VCd. Abbreviations as in Figure 1.

Rates of overall hematologic CR and 6-month cardiac and renal response were all higher with D-VCd vs VCd in the overall study population and across all cardiac stages (Figure 1). Among patients who were evaluable for cardiac response, the rate of cardiac progression at 6 months was numerically lower in the D-VCd group compared with the VCd group (13.6% [95% CI: 8.0%-21.1%] vs 19.7% [95% CI: 12.9%-28.0%]). Because the number of patients who experienced cardiac progression at 6 months was small, this was not analyzed by cardiac stage. Both major organ deterioration–PFS and major organ deterioration–EFS were prolonged in the D-VCd group compared with the VCd group, across all cardiac stages (Figure 2). Within each treatment group, there was a trend toward longer major organ deterioration–PFS and major organ deterioration–EFS in patients with less severe baseline cardiac stage.

The incidence of cardiac events (all grades) was numerically higher with D-VCd compared with VCd (overall and during cycles 1-6), but the incidence of grade 3 or 4 events was similar between groups (Table 2). More patients in the D-VCd group than in the VCd group had cardiac events in the first month of the study (Supplemental Figure 1). The most common cardiac events included cardiac failure, atrial fibrillation, and palpitations (the latter likely a consequence of atrial fibrillation and other arrythmias associated with cardiac failure). For further insight into the nature of the cardiac events during the study, AEs were analyzed by cardiac involvement and stage. Most patients in both treatment arms had cardiac involvement (Table 3). The incidence of AEs of any grade was similar between treatments and by cardiac involvement (Table 3); the incidence of grade 3 or 4 and serious AEs (SAEs) was higher in patients with cardiac involvement than in those without. All cardiac SAEs in the D-VCd arm (32 of 32 patients) and most cardiac SAEs in the VCd arm (24 of 25 patients) occurred in patients with cardiac involvement (Table 3); the number of deaths among patients with baseline cardiac involvement was numerically higher with D-VCd (23 of 140) than with VCd alone (16 of 133), and all fatal cardiac events occurred in patients with cardiac involvement (D-VCd, 15 of 15; VCd, 8 of 8). Baseline medical histories were manually reviewed for underlying patterns predictive of the observed cardiac AEs. Among the 62 assessed patients with cardiac AEs of any grade (D-VCd, n = 42; VCd, n = 20), 26 had medical histories of ≥1 cardiac disorder, including cardiac failure (n = 20), atrial fibrillation (n = 6), and palpitations (n = 4). No history of cardiac failure, atrial fibrillation, or palpitations was reported in the other 36 patients.

**Table 2:** Incidence of Cardiac Events by Treatment Group

	D-VCd						VCd			
	All Grades			Grade 3 or 4			All Grades		Grade 3 or 4	
	All (N = 193)	Cycles 1-6	Cycles 7 and Later	All (N = 193)	Cycles 1-6	Cycles 7 and Later	All (N = 188)	Cycles 1-6	All (N = 188)	Cycles 1-6
≥1 cardiac event^a^	67 (36.9)	58 (30.8)	20 (15.9)	22 (11.7)	21 (11.1)	4 (3.4)	41 (27.1)	41 (22.8)	18 (10.6)	18 (10.2)
Cardiac events^a^										
Cardiac failure^b^	17 (8.9)	17 (8.9)	2 (1.4)	12 (6.3)	12 (6.3)	1 (0.7)	14 (7.8)	14 (7.8)	9 (5.1)	9 (5.1)
Atrial fibrillation	12 (6.8)	9 (4.8)	3 (2.5)	3 (1.6)	3 (1.6)	0	4 (2.9)	4 (2.4)	1 (0.5)	1 (0.5)
Palpitations	11 (6.3)	9 (4.8)	3 (2.4)	0	0	0	6 (4.9)	6 (3.5)	0	0

Values are n (%). Cycles 1 to 6 and cycles 7 and later groups are not mutually exclusive.

Abbreviations as in Table 1.

a Fine and Gray’s method was used to assess the cumulative incidence rate of cardiac events (including cardiac failure, atrial fibrillation, and palpitations) by considering death as a competing event for the first cardiac event.

b Includes cardiac failure and cardiac failure congestive.

**Table 3:** Adverse Events and Serious Adverse Events by Cardiac Involvement

	D-VCd			VCd		
	Baseline Cardiac Involvement			Baseline Cardiac Involvement		
	Yes (n = 140)	No (n = 53)	Total (N = 193)	Yes (n = 133)	No (n = 55)	Total (N = 188)
Any AE	138 (98.6)	51 (96.2)	189 (97.9)	132 (99.2)	53 (96.4)	185 (98.4)
≥1 grade 3 or 4 AE	89 (63.6)	26 (49.1)	115 (59.6)	81 (60.9)	27 (49.1)	108 (57.4)
≥1 SAE	72 (51.4)	15 (28.3)	87 (45.1)	57 (42.9)	11 (20.0)	68 (36.2)
Cardiac SAEs^a^	32 (23.4)	0	32 (17.1)	24 (20.7)	1 (2.1)	25 (15.2)
Cardiac failure^b^	13 (9.3)	0	13 (6.8)	10 (8.0)	0	10 (5.6)
Cardiac arrest	8 (5.9)	0	8 (4.3)	3 (2.7)	0	3 (1.9)
Atrial fibrillation	5 (3.8)	0	5 (2.8)	2 (1.9)	0	2 (1.4)
Deaths	23 (16.4)	1 (1.9)	24 (12.4)	16 (12.0)	0	16 (8.5)
Deaths due to cardiac events	15 (10.7)	0	15 (7.8)	8 (6.0)	0	8 (4.3)

Values are n (%). The total number of patients with cardiac SAEs includes additional cardiac events not reported here.

AE = adverse event; SAE = serious adverse event; other abbreviations as in Table 1.

a Fine and Gray’s method was used to assess the cumulative incidence rate of cardiac SAEs (including cardiac failure, cardiac arrest, and atrial fibrillation) by considering death as a competing event for the first cardiac event.

b Includes cardiac failure and cardiac failure congestive.

Further analysis of AEs by cardiac stage demonstrated that rates of AEs of any grade were similar across treatment groups and cardiac stages (Table 4). Within each treatment group, rates of SAEs increased with worsening cardiac stage. Most patients who experienced serious or fatal cardiac events had baseline cardiac stage II or IIIA or baseline NYHA functional class II or IIIA. These data suggest that most cardiac-related deaths were attributable to the underlying AL amyloidosis–related cardiomyopathy.

**Table 4:** AEs and Cardiac AEs by Cardiac Stage and NYHA Functional Class

	D-VCd			VCd		
	Cardiac Stage			Cardiac Stage		
	I	II	IIIA	I	II	IIIA
Any-grade AE	44 (95.7)	74 (98.7)	71 (98.6)	40 (95.2)	79 (100.0)	66 (98.5)
SAE	10 (21.7)	32 (42.7)	46 (62.5)	7 (16.7)	25 (31.6)	36 (53.7)
≥1 grade 5 or serious cardiac AE	0	10 (13.6)	22 (31.1)	1 (2.8)	7 (10.3)	17 (28.6)
Atrial fibrillation	0	1 (1.3)	4 (6.0)	0	0	2 (3.9)
Cardiac arrest	0	2 (2.6)	6 (8.6)	0	2 (2.6)	1 (1.8)
Cardiac failure	0	3 (4.0)	10 (13.8)	0	1 (1.3)	9 (14.3)
≥1 grade 5 cardiac AE	0	4 (5.3)	11 (15.7)	0	3 (3.8)	5 (9.0)
Cardiac arrest	0	1 (1.3)	6 (8.6)	0	2 (2.6)	1 (1.8)
Cardiac failure	0	1 (1.3)	4 (5.8)	0	0	2 (3.8)

	NYHA Functional Class			NYHA Functional Class		
	I	II	IIIA	I	II	IIIA
Any-grade AE	95 (96.0)	77 (100.0)	17 (100.0)	91 (97.8)	85 (100.0)	9 (90.0)
SAE	3 (3.0)	21 (27.3)	8 (47.1)	6 (6.5)	16 (18.8)	3 (30.0)
≥1 grade 5 or serious cardiac AE^a^	3 (3.1)	21 (27.8)	8 (48.5)	6 (6.8)	16 (22.2)	3 (32.7)
Atrial fibrillation	0	5 (7.0)	0	1 (1.1)	1 (1.5)	0
Cardiac arrest	1 (1.0)	4 (5.4)	3 (17.7)	0	2 (3.2)	1 (9.8)
Cardiac failure^b^	2 (2.0)	7 (9.1)	4 (23.6)	2 (2.2)	7 (8.5)	1 (11.7)
≥1 grade 5 cardiac AE^a^	2 (2.2)	8 (10.5)	5 (29.6)	2 (3.6)	4 (5.5)	2 (20.2)
Cardiac arrest	1 (1.0)	3 (4.0)	3 (17.7)	0	2 (3.3)	1 (9.8)
Cardiac failure^b^	1 (1.1)	3 (3.9)	1 (5.9)	1 (3.3)	0	1 (10.5)

Values are n (%). The total number of patients with ≥1 grade 5 or serious cardiac AE and ≥1 grade 5 cardiac AEs includes additional cardiac events not reported here.

Abbreviations as in Tables 1 and 3.

a Fine and Gray’s method was used to assess the cumulative incidence rate of cardiac events (≥1 grade 5 or serious cardiac AEs including atrial fibrillation, cardiac arrest, and cardiac failure and ≥1 grade 5 cardiac AE including cardiac arrest and cardiac failure) by considering death as a competing event for the first cardiac event.

b Includes cardiac failure and cardiac failure congestive.

Given the observed difference in treatment duration between the D-VCd and VCd arms (13.4 months vs 5.3 months), we analyzed the exposure-adjusted AE incidence rates and found that the incidence of all reported AEs was lower for D-VCd vs VCd (Table 5). Additionally, exposure-adjusted incidence rates for any-grade and grade 3 or 4 cardiac AEs were lower with D-VCd vs VCd (3.48 vs 5.47 for any grade, 0.97 vs 2.25 for grade 3 or 4 [data not shown]).

**Table 5:** Summary of Exposure-Adjusted Adverse Event Incidence Rates

	D-VCd			VCd		
	n	100 Patient-Months at Risk^a^	EAIR^b^	n	100 Patient-Months at Risk^a^	EAIR^b^
n	193			188		
Any TEAE	189	1.37	137.46	185	0.85	217.92
≥1 related	174	3.02	57.67	169	1.81	93.32
Maximum toxicity grade						
Grade 1	7	23.30	0.30	10	7.89	1.27
Grade 2	61	17.52	3.48	61	6.51	9.38
Grade 3	78	16.11	4.84	83	6.72	12.35
Grade 4	19	22.71	0.84	15	8.00	1.88
Grade 5	24	23.99	1.00	16	8.25	1.94

EAIR = exposure-adjusted incidence rate; TEAE = treatment-emergent adverse event; other abbreviations as in Tables 1 and 3.

a Patient-months at risk is the sum of the exposure times at the occurrence of the first TEAE for each subject. A patient’s duration of exposure is given either by the time when the event occurred (noncensored data) or by the total duration of treatment if the patient does not show the AE in question (censored data).

b EAIR represents the number of subjects with the event divided by the 100 patient-months at risk for that event. If a patient has multiple occurrences of an event, the patient is counted only once in the numerator.

**Figure 3:**
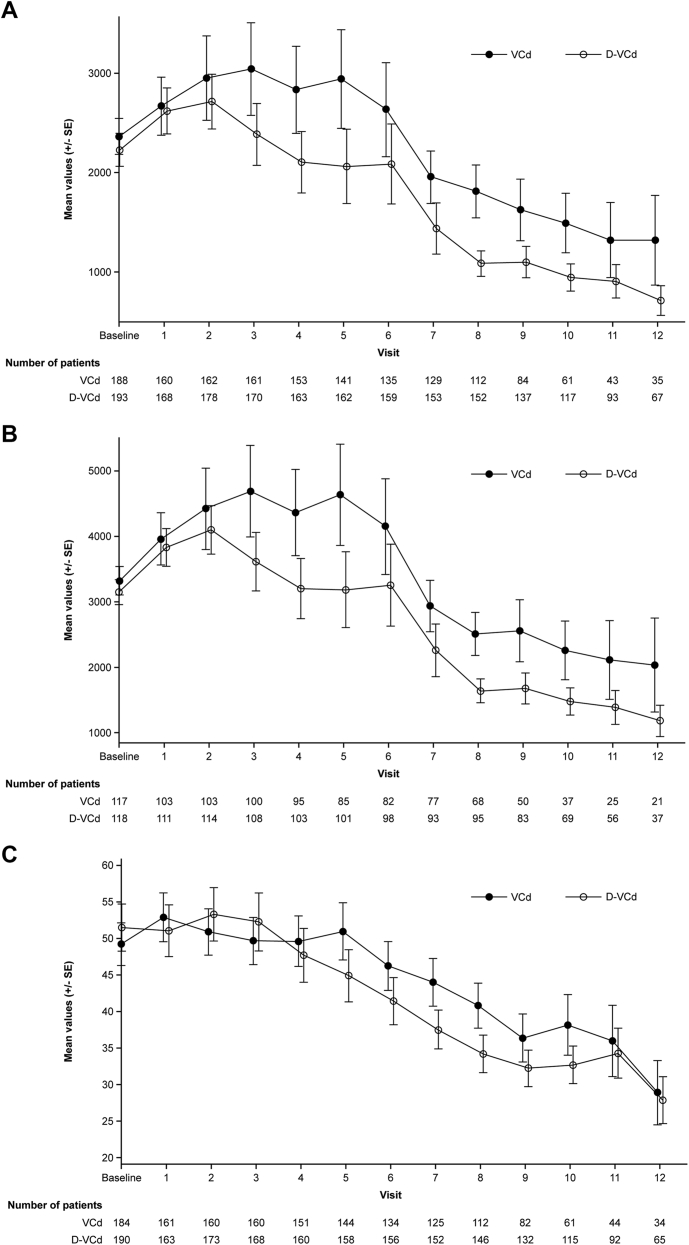
NT-proBNP in ITT Analysis Set, Patients Evaluable for Cardiac Response, and Troponin Mean N-terminal pro–brain natriuretic peptide (NT-proBNP) in the ITT population **(A)** and in cardiac response-evaluable patients **(B)** and mean high-sensitivity troponin levels **(C)** decreased over time in both groups, suggesting no negative impact on cardiac function from the addition of daratumumab to VCd. Disease evaluations were conducted at the screening phase, during treatment cycles 1-6, and every 8 weeks until disease progression. Abbreviations as in Figure 1.

In the intent-to-treat population and in patients who were evaluable for cardiac response, NT-proBNP levels increased during the first treatment cycles, followed by a decline over time, which was more pronounced and occurred earlier with D-VCd vs VCd (Figure 3). Mean high-sensitivity troponin remained stable during initial treatment; thereafter, it gradually decreased over time in both treatment arms.

## Discussion

Earlier studies have explored the use of high-dose melphalan and autologous stem cell transplantation in patients with AL amyloidosis. Although this treatment combination induced good hematologic and organ response in patients, it was associated with a high rate of mortality if patients were not carefully selected and thus was not recommended for high-risk patients, especially those with advanced cardiac involvement.^22^ The advent of novel agents has improved long-term outcomes in AL amyloidosis overall, but few studies highlight such benefits in high-risk patients.^16^ ANDROMEDA included stage IIIA patients but excluded the highest risk group (stage IIIB). As about 70% of patients in ANDROMEDA had cardiac involvement and one-third were in cardiac stage IIIA, in this post hoc analysis we examined the efficacy and safety outcomes in patients with amyloidosis by cardiac stage.

The primary study results demonstrated the superiority of D-VCd over VCd alone in newly diagnosed AL amyloidosis.^17^ On the basis of these results, D-VCd became the first treatment for AL amyloidosis to receive regulatory approval. During the approval process, potential cardiotoxicity of D-VCd in patients with AL amyloidosis was raised as a concern by the regulatory authorities. Hence, we assessed the rates of overall hematologic CR and 6-month cardiac and renal response in patients with cardiac stage I, II, or IIIA in this analysis. Major organ deterioration–PFS and major organ deterioration–EFS were also assessed across cardiac stages. Moreover, we explored the cardiac safety of D-VCd vs VCd across different cardiac stages, which was not analyzed in the primary study. In this study, more patients in the D-VCd group achieved overall hematologic CR (Central Illustration) and 6-month organ responses than in the VCd group. The difference between treatment groups was more pronounced in the stage IIIA subgroup (61.1% vs 10%; OR: 14.14; 95% CI: 5.67-35.25) for cardiac response, suggesting that these high-risk patients, who may have poor clinical response and higher rates of death, may be particularly likely to benefit from D-VCd. It is very likely that in the long term, these high-risk patients may show improvements in cardiac function and overall survival. Although the 6-month renal response rate was higher in patients treated with D-VCd vs VCd, the response rate was lower in patients in cardiac stage IIIA than those in cardiac stages I and II. This may be due to estimated glomerular filtration rate deterioration in these high-risk patients, which may have affected the renal response. Regardless of cardiac stage, major organ deterioration–PFS and major organ deterioration–EFS were longer with D-VCd than VCd (Central Illustration). Irrespective of treatment, there was a tendency toward longer major organ deterioration–PFS and major organ deterioration–EFS in patients with less severe cardiac stage (likely because of the higher rates of cardiac death in patients with more advanced cardiac stage at baseline).

**Central Illustration:**
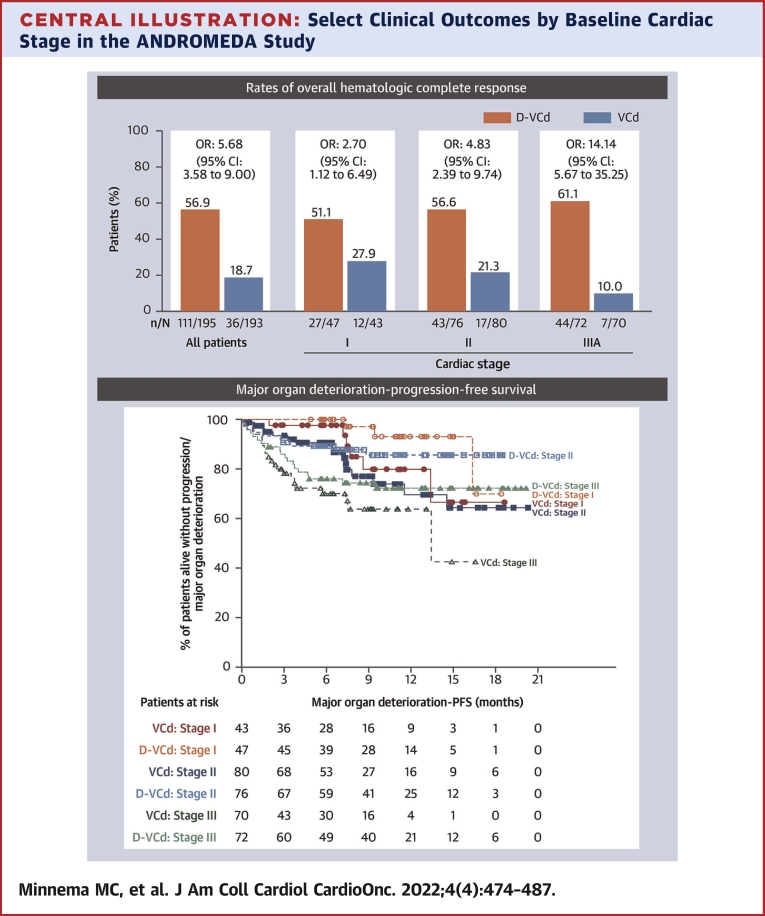
Select Clinical Outcomes by Baseline Cardiac Stage in the ANDROMEDA Study Rates of overall hematologic complete response and major organ deterioration–progression-free survival were improved with daratumumab, bortezomib, cyclophosphamide, and dexamethasone (D-VCd) compared with bortezomib, cyclophosphamide, and dexamethasone (VCd) in patients with newly diagnosed amyloid light chain amyloidosis.

Several studies have assessed clinical outcomes in AL amyloidosis by cardiac stage, although cross-trial comparisons should be interpreted with caution because of differences in eligibility criteria, study design, definitions of study endpoints, and the time points at which they were assessed. In retrospective analysis of 230 patients treated with VCd, 29% had baseline cardiac stage IIIA and 20% had stage IIIB.^6^ Rates of hematologic CR (ie, normal FLC ratio and negative serum and urine immunofixation) in 201 patients with measurable disease and cardiac stages I, II, IIIA, and IIIB were 33%, 18%, 23%, and 14%, respectively. Cardiac response rates were 29%, 17%, and 4% in stages II, IIIA, and IIIB, respectively. These findings are consistent with the hematologic CR and 6-month cardiac response observed in the VCd group of our study, except that the proportion of patients in cardiac stage IIIA was high in our study (36.6%).

Manwani et al^12^ reported outcomes in 915 patients with newly diagnosed AL amyloidosis treated with bortezomib-based regimens. The proportion of patients with cardiac stage IIIA in that study (37.6%) was comparable with that in our study (36.6%). The investigators reported that 38% of evaluable stage III patients achieved hematologic CR at 6 months, which was much higher than that reported in our study for patients in the VCd group. Another retrospective analysis (N = 60) examining VCd in the front line reported an overall hematologic response rate of 68% in patients with cardiac stage III, with 10 patients (17%) achieving hematologic CR, comparable with that seen in cardiac stage IIIA patients treated with VCd in our study. However, survival outcomes in patients in stage IIIB were poor (median survival 4.4 months).^23^ For patients treated with D-VCd in our study, although longer follow-up is required to determine survival outcomes, rates of hematologic CR across cardiac stages (stage I, 51.1%; stage II, 56.6%; stage IIIA, 61.1%) were higher than among those treated with VCd.

In the phase 3 EMN-03 study (N = 109), for which stage IIIB patients were ineligible, rates of hematologic CR and 9-month cardiac and renal responses favored bortezomib, melphalan and dexamethasone vs melphalan, and dexamethasone (23% vs 20%, 38% vs 28%, and 33% vs 26%, respectively) in patients with AL amyloidosis.^13^ Although these data are for the intent-to-treat population, a similar trend in terms of favorability was observed for rates of any hematologic response across cardiac stages in these patients, with a statistically significant difference seen for patients with cardiac stage II (78% vs 51%; *P* = 0.010). In our study, hematologic CR and cardiac and renal responses at 6 months favored D-VCd over VCd across all cardiac stages.

In our study, rates of SAEs were higher in patients with cardiac involvement and more advanced cardiac stage, regardless of treatment. Although the number of deaths among patients with cardiac involvement at baseline and the rate of cardiac events were numerically higher with D-VCd vs VCd, evidence suggests that this is likely due to underlying AL amyloidosis–related cardiomyopathy rather than daratumumab treatment. Although there are important differences between patients with multiple myeloma and those with AL amyloidosis, the robust body of evidence from clinical trials of daratumumab in multiple myeloma can provide general insights into its safety profile. Previous studies have not indicated an association between daratumumab and serious cardiac toxicity in patients with newly diagnosed or relapsed multiple myeloma.^24^, ^25^, ^26^, ^27^ Cardiac SAEs in the present study were observed almost exclusively in patients with cardiac involvement in both treatment groups. Case-level reviews failed to demonstrate an observed or apparent cardiac or cardiovascular baseline pattern associated with increased risk for developing cardiac AEs during the study, and exposure adjustment corrected for the observed higher frequency of overall AEs, SAEs, and grade 5 AEs with D-VCd. Last, NT-proBNP and high-sensitivity troponin levels decreased over time in both groups, but this occurred faster and reached lower levels with D-VCd. As these parameters would be expected to increase in patients treated with a cardiotoxic regimen, this observation further supports the idea that the observed cardiac events are likely due to the underlying disease and that the addition of daratumumab to VCd has no deleterious effect on cardiac function and is well tolerated in patients. Moreover, these biomarker responses were positively associated with the hematologic response achieved by these patients. Longer follow-up is needed to determine the improvement in cardiac response and survival outcomes in these patients.

We had hoped to see a reduction in the rate of early deaths with D-VCd. Although we found that similar numbers of patients died in both groups at 1 year, most deaths occurred during the first 6 months of treatment, likely related to irreversible cardiac damage present at baseline. Patients who survive beyond the first 6 months may therefore have the opportunity to benefit from D-VCd and achieve hematologic CR. It is critical to provide the best possible care with the most effective treatment regimens as early as possible, to halt organ damage and improve prognosis.^28^^,^^29^ The rapid, deep hematologic responses observed with D-VCd^30^ support its use as a novel standard of care for initial treatment of patients with AL amyloidosis. An ongoing phase 2 study (NCT04131309) is evaluating daratumumab monotherapy in patients with stage IIIB AL amyloidosis and is expected to provide further evidence regarding the efficacy and safety of daratumumab in patients with severe cardiac involvement.

### Study limitations

The analyses were not preplanned. Although baseline characteristics were largely similar across stages I, II, and IIIA, some differences were observed with increasing cardiac stage (older age, worse Eastern Cooperative Oncology Group performance status, more advanced renal failure, and increased difference between involved and uninvolved FLC). Although these attributes are likely reflective of the higher disease burden in patients in higher cardiac stages, they may also have independently affected the outcomes reported here. Our study lacks detailed information on the nature of arrhythmias during the cardiac events. Because of the exploratory nature of the analyses, no formal statistical comparisons were conducted. Longer follow-up is needed to assess late cardiac response (∼12 months) and determine whether these findings are associated with differences in overall survival.

## Conclusion

These results demonstrate that D-VCd benefits patients with newly diagnosed AL amyloidosis in both hematologic and organ responses across cardiac stages I to IIIA.

Perspectives **COMPETENCY IN MEDICAL KNOWLEDGE:** Severe cardiac involvement at baseline is associated with poor prognosis in patients with AL amyloidosis. Patients treated with D-VCd experienced better clinical outcomes than those treated with VCd, irrespective of the severity of cardiac involvement, supporting its use in a broad range of patients.

**TRANSLATIONAL OUTLOOK:** Future research should examine efficacy and safety outcomes of treatment, including D-VCd, among the most high-risk patients (stage IIIB), with special attention to cardiac outcome parameters.

## Funding Support and Author Disclosures

This research was funded by Janssen Research & Development. Prof Minnema has consulted or served in an advisory role for Janssen-Cilag, Alnylam, and Gilead; has served on a Speakers Bureau for Bristol Myers Squibb; and has received travel, accommodation, and expense compensation from Celgene. Dr Dispenzieri has received research funding from Alnylam, Celgene, Intellia, Janssen, Pfizer, and Takeda. Dr Comenzo has consulted or served in an advisory role for Amgen, Caelum, Janssen, Karyopharm, Prothena, Sanofi, Takeda, and Unum; and has received research funding from Janssen, Karyopharm, Prothena, and Takeda. Dr Kastritis has consulted or served in an advisory role for and has received honoraria from Amgen, Genesis Pharma, Janssen, Pfizer, and Takeda; and has received research funding from Amgen and Janssen. Prof Wechalekar has consulted or served in an advisory role for Caelum and Janssen; has received honoraria from Celgene, Janssen, and Takeda; and has received travel, accommodation, and expense compensation from Takeda. Dr Witteles has served in an advisory role for Alnylam Pharmaceuticals, Pfizer, Akcea/Ionis, Eidos, and Regeneron. Dr Ruberg has consulted for Attralus and Alexion; and has received research funding from Akcea, Alnylam, and Pfizer. Dr Maurer has consulted or served in an advisory role for Alnylam Pharmaceuticals and Pfizer; and has received research funding from Akcea, Alnylam Pharmaceuticals, Eidos, Ionis, Intellia, Novo Nordisk, and Pfizer. Prof Jaccard has consulted or served in an advisory role for Janssen; and has received honoraria, research funding, and travel, accommodation, and expense compensation from Celgene and Janssen. Dr Weiss was an employee of Janssen at the time the study was conducted. Dr Tran, Mr Qin, Ms Vasey, and Dr Vermeulen are employees of Janssen. All other authors have reported that they have no relationships relevant to the contents of this paper to disclose.
