# Supplementary material for: Outcomes by Cardiac Stage in Patients With Newly Diagnosed AL Amyloidosis: Phase 3 ANDROMEDA Trial
Source: JACC CardioOncol. 2022 Nov 15;4(4):474–87. doi: 10.1016/j.jaccao.2022.08.011 (PMC9700253; doi:10.1016/j.jaccao.2022.08.011)

# SUPPLEMENTAL APPENDIX

**Outcomes by Cardiac Stage in Patients With Newly Diagnosed AL Amyloidosis: Phase 3 ANDROMEDA Trial**

Monique C. Minnema, MD^a^; Angela Dispenzieri, MD^b^; Giampaolo Merlini, MD^c,d^; Raymond L. Comenzo, MD^e^; Efstathios Kastritis, MD^f^; Ashutosh D. Wechalekar, MD^g^; Martha Grogan, MD^b^; Ronald Witteles, MD^h^; Frederick L. Ruberg, MD^i^; Mathew S. Maurer, MD^j^; NamPhuong Tran, MD^k^; Xiang Qin, MS^l^; Sandra Y. Vasey, MS^l^; Brendan M. Weiss, MD^l^; Jessica Vermeulen, MD, PhD^m^; Arnaud Jaccard, MD, PhD^n^

From the ^a^University Medical Center Utrecht, Utrecht, Netherlands; ^b^Mayo Clinic, Rochester, Minnesota, USA; ^c^Amyloidosis Research and Treatment Center, Fondazione IRCCS Policlinico San Matteo, Pavia, Italy; ^d^Department of Molecular Medicine, University of Pavia, Pavia, Italy; ^e^John C Davis Myeloma and Amyloid Program, Tufts Medical Center, Boston, Massachusetts, USA; ^f^National and Kapodistrian University of Athens, Athens, Greece; ^g^University College London, London, UK; ^h^Stanford Amyloid Center, Stanford University School of Medicine, Stanford, California, USA; ^i^Boston Medical Center, Boston University School of Medicine, Boston, Massachusetts, USA; ^j^Columbia University Irving Medical Center, New York, New York, USA; ^k^Janssen Research & Development, LLC, Los Angeles, California, USA; ^l^Janssen Research & Development, LLC, Spring House, Pennsylvania, USA; ^m^Janssen Research & Development, LLC, Leiden, Netherlands; ^n^Centre Hospitalier Universitaire and Reference Center for AL Amyloidosis, Limoges, France.

**SUPPLEMENTAL FIGURE 1 Fine and Gray estimates of (A) time to first cardiac event and (B) time to first grade 3/4 cardiac event.**

A time-to-event analysis revealed that a greater number of patients in the D-VCd group had cardiac events in the first month of the study compared with those in the VCd group
D-VCd = daratumumab, bortezomib, cyclophosphamide, and dexamethasone; VCd bortezomib = cyclophosphamide, and dexamethasone.

Fine and Gray’s method was used to assess time to first cardiac event and time to first grade 3/4 cardiac event.


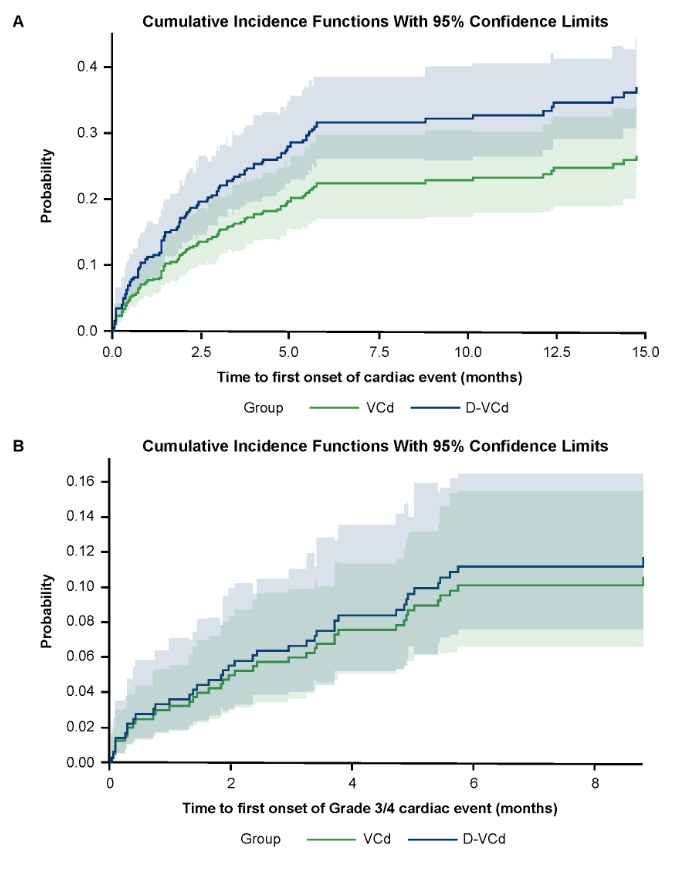

Supplement: Supplemental Figure 1 [file mmc1.docx]
